# Lifestyle in Female Teachers: Educational Intervention Based on Self-Efficacy Theory in the South of Fars Province, Iran

**DOI:** 10.1155/2021/6177034

**Published:** 2021-12-06

**Authors:** Fariba Abbasi, Leila Ghahremani, Mahin Nazari, Mohammad Fararouei, Zakieh Khoramaki

**Affiliations:** Research Center for Health Sciences, Institute of Health, Department of Health Promotion, School of Health, Shiraz University of Medical Sciences, Shiraz, Iran

## Abstract

**Introduction:**

Today, improving lifestyles and promoting health are basic needs for human society. The main goal in promoting health is to achieve healthy lifestyle behaviors, and self-efficacy is one of the factors influencing people's lifestyle. Therefore, the impact of educational intervention based on self-efficacy theory on improving lifestyles of the female teachers in Galledar was investigated.

**Method:**

This study was a semiexperimental study with educational intervention with a control group that was performed on 120 teachers in Galledar. Data collection tools included demographic information questionnaires, health-promoting lifestyle questionnaires, and Sherry's self-efficacy questionnaire. Data were analyzed using SPSS 25 software.

**Result:**

The mean age and standard deviation of teachers in the control and intervention groups were 33.40 ± 5.68 and 32.83 ± 6.46 years, respectively. Health-promoting lifestyle variables are significant correlation with self-efficacy and overall lifestyle index. Six dimensions which consisted of spiritual growth and self-actualization, health responsibility, interpersonal relationships, stress management, exercise and physical activity, and nutrition showed significant statistical differences before and after educational intervention (*P* = 0.001).

**Conclusion:**

Due to the sensitive role of teachers as an effective human force in the development and evolution of society and their students' role modeling, the authorities should formulate policies, regulate educational interventions, and design strategies for promoting self-efficacy beliefs and promoting a healthy lifestyle for all teachers. We suggest that other methods and theories of behavior change be used in future studies to promote a healthy lifestyle.

## 1. Introduction

Health is one of the most important and basic human needs and a tool to achieve the goals and successes of individuals and communities that must be planned throughout life to maintain health [1]. One of the determinants of people's health is their lifestyle [2].

Lifestyle is a relatively coherent set of all a person's behaviors and activities during life [3]. A health-promoting lifestyle helps maintain and strengthen a person's level of health and self-actualization. It also leads to optimal well-being, individual development, and creative life, and it has six dimensions of interpersonal relationships, health responsibility, spiritual growth and self-actualization, stress management, nutrition, and physical activity [4].

More than a third of deaths worldwide are due to poor personal and social lifestyles. The World Health Organization said, in a statement at the first World Conference on Healthy Lifestyle in 2011 in Moscow, that 60% of global deaths and 80% of deaths in developing countries are currently due to unhealthy lifestyles. The share of this factor in global mortality will reach to 75% by 2030 [5], and one of the main reasons for the increase in noncommunicable diseases is the adoption of unhealthy behaviors, too [6].

Adopting healthy behaviors has a significant impact on reducing health costs, mortality, and life stressors, as well as increasing life expectancy and improving the quality of life of individuals, which have a special importance and place in society [7, 8]. About 80% of women aged 18-55 have high-risk, lifestyle-related behaviors that are preventable [9]. However, the results of a study by Tamakoshi et al. showed that 18.5% of deaths among women can be prevented if lifestyle management is managed [10].

Evidence suggests that self-efficacy is related to lifestyle [11, 12]. It is one of the factors influencing the lifestyles of individuals and shows people's confidence and confidence in their skills and ability to successfully perform behavior in certain situations [13–15]. Studies have shown that self-efficacy is associated with a variety of health-promoting lifestyle behaviors, such as smoking cessation, diet, and physical activity [16, 17]. It also leads to better self-management, increased life expectancy, and adjusted health behaviors [18], and it is one of the factors that lead to the promotion of women's general health [19]. There are four major sources of self-efficacy: performance gains (such as previous successful experiences), succession experiences (such as using a successful model), verbal encouragement (encouragement from influential people such as friends and family), and emotional arousal. [15, 20–22]. According to the results of a study by Zeldin et al. on women's self-efficacy beliefs in math, science, and technology, self-efficacy leads to the endurance and flexibility needed to overcome scientific and professional barriers [23].

One of the ways to achieve the goal of following up on diseases is to use appropriate educators for the target community. Meanwhile, the role of teachers as facilitators of general education and the basic role models of students [24] are very important [22].

A female teacher is responsible for a dual role, one in the family and the other in the work [25]. During their daily work, school teachers face a variety of stressors (for example, noise in the classroom, problems with students and their parents, and extra administrative work) that lead to significant chronic stress [22]. However, teachers are the main pillar of education, and female teachers are also responsible for raising children at home and play an important role in educating the next generation of the country [9, 22]. They need to have the necessary skills and abilities to play their role in the best possible way, because the main burden of educating students is on teachers [25]. By adhering to healthy behaviors and adopting a healthy lifestyle, they can be an effective role model for their families and students [26].

The results of a study by Charkazi et al. showed that 84% of teachers working in Gorgan schools had a semifavorable lifestyle [27]. In their study, Pirzadeh et al. found that 14.5% of teachers had a semidesirable and desirable lifestyle, of which 26.1% was related to nutrition, 76% to physical activity, and 29.2% to smoking or exposure. And 21.9% had a semioptimal lifestyle to deal with stress [26].

Health-promoting lifestyle training is one of the most important factors in increasing knowledge and self-efficacy [28] and improving women's performance to improve health [29]. The effectiveness of health education programs largely depends on the correct use of the theories and models used in it, and intervention programs based on a theoretical framework can increase the individual's understanding of the factors affecting behavior change such as psychological and social causes and biology, to facilitate the process of changing health behaviors [30].

Therefore, considering the importance of lifestyle variables in promoting health and increasing quality of life, the sensitive role of teachers as an influential human force in the development and evolution of society and students' role models [24, 27] as well as the uncertainty of the role of self-efficacy in health-promoting lifestyle [31], and finally the lack of interventional studies on teachers' lifestyle, this study was conducted to investigate the effect of educational intervention based on self-efficacy theory on improving the lifestyle of female teachers in Galledar in the south of Fars province.

## 2. Materials and Methods

This study was a semiexperimental intervention research conducted in 2018-19 with the aim of investigating the effect of educational intervention based on self-efficacy theory on improving lifestyles of all female teachers in Galledar located in Fars province.

The sample size of this study was estimated based on similar previous studies [32], and considering the formula for the average difference between the two groups, as well as the first type of error of 5% and the test power of 80%, we estimated 48 people for each group. The drop ranged from 20% to 60% in each group.

The statistical population of this study included all female teachers working in Galledar in 2018-19. There were a total of six girl schools, four elementary schools, and two junior high schools in the city. With the two-step sampling method, we first divided the schools into primary and junior high schools, and then, we randomly assigned two primary schools and one junior high school to the test group, as well as two primary schools and one junior high school to the control group. Then, from the list of teachers of the mentioned schools, we selected the desired samples using simple random sampling.

Inclusion criteria for participating in the study were being a female, being a teacher, residing in the city of Galledar, having a continuous and consecutive attendance at the training, willing to participate in the study, and completing the conscious consent form. Individuals were excluded from the study if they changed their place of residence outside of the research environment, had more than one absence session in the training classes, or did not attend one of the pretest or posttest stages.

### 2.1. Research Tools

- The demographic variable questionnaire included age, marital status, level of education, number of deliveries, tobacco use, spouse's education rate, spouse's job, and average household income
- The health-promoting lifestyle questionnaire (Walker et al. 1987) measures a health-promoting lifestyle by focusing on initiatives and individual perception of self-improvement, self-fulfillment, and individual satisfaction. It consists of 54 items with six dimensions of nutrition with 11 questions, physical activity with 13 questions, responsibility in health with 8 questions, stress management with 6 questions, interpersonal relationships with 8 questions, and spiritual growth and self-actualization with 8 questions. Each item in this questionnaire is given a score of one (never) to four (always). The overall score of health-promoting behaviors is in the range of 52 to 208, and a separate score is calculable for each dimension. Mohamadi conducted a study in Iran. He reported the Cronbach alpha coefficient for the entire tool at 0.82 and for the subbranches in the range of 0.64 to 0.91 [33]
- Sherry general self-efficacy questionnaire (1982) includes 17 items and is graded on a 1-5 Likert scale to measure self-efficacy in doing things. In this scale, scores of 1 to 5 from right to left belong to questions 15, 13, 9, 8, 3, and 1, and the rest of the questions are scored in reverse, i.e., from left to right. Thus, the highest efficacy score on this scale is 85 and the lowest is 17. Barati has translated and presented the Persian version of this questionnaire [34]

In order to conduct the research, we firstly obtained a license from the research deputy of Shiraz University of Medical Sciences, and then, we made the necessary coordination with the Department of Education of the Galledar region. In the next step, the teachers included in the present study were justified on how we plan and keep the information confidential. Then, we collected the information and distributed the questionnaires before the educational intervention in both groups.

We developed educational content based on authoritative sources in six dimensions of healthy lifestyle using self-efficacy theory structures, which are described in Table 1. We taught six 40- to 45-minute training sessions through interactive lecturing, group discussion, and the use of media such as educational pamphlets and slide shows as part of the PowerPoint program.

In order to enhance the self-efficacy of the participants, they were asked to share their successful experiences with others. To this end, one of the teachers who had implemented effective methods of stress management was invited to present his experiences and answer questions (mastery experiences and role models) so that the learners who had participated in the discussion for verbal persuasion were encouraged. Individual counseling was also conducted to reinforce participants who were not very successful in implementing health-promoting behaviors, and they were asked to break down their goals into smaller, more achievable steps and were successfully encouraged after each step. Sending persuasive text messages also encouraged the participants. Participants were also allowed to express feedback on their thoughts, feelings, and experiences in performing physical activity, adhering to a healthy diet, and performing stress management techniques. Individual counseling was also provided by a psychologist to participants who had difficulty controlling their emotions, moods, and physiology.

It should be noted that throughout the intervention, the teachers of the control group were unaware of the implementation of the relevant educational program.

Two months after the implementation of the educational intervention, we performed posttests on both the test and control groups of teachers using the same initial questionnaires.

We analyzed the collected data using SPSS statistical software version 25. In addition to descriptive analysis, we used the chi-square test and independent *t*-test to compare the variables of the two groups and paired *t*-test for the intragroup comparison.

## 3. Results

A total of 120 teachers participated in the study. The mean age and standard deviation of teachers in the control and intervention groups were 33.40 ± 5.68 and 32.83 ± 6.46 years, respectively; there was no significant difference in age between these two groups (*P* value = 0.61). In Table 2, we presented information on participants in the two groups of intervention and control in terms of age, average number of deliveries, and average service history by year and average daily physical activity of teachers. The intervention and control groups were homogeneous in terms of the three variables mentioned and were not significantly different (Table 3).

We have provided the correlation coefficient of the research variables in Table 4. The results show that all health-promoting lifestyle variables are related to each other and to the overall lifestyle index, and they also have a significant correlation with self-efficacy.

## 4. Discussion and Conclusion

The aim of this study was to investigate the effect of education based on self-efficacy theory on improving the lifestyle of female teachers. The results of this study show that the overall score of the health-promoting lifestyle in the intervention group increased significantly after two months of the training program. Therefore, the overall score of lifestyles in the intervention group increased from 137.53 ± 9.41 to 155.68 ± 24.58; this indicates the positive effect of the training program.

Six dimensions of spiritual growth and self-actualization, health responsibility, interpersonal relationships, stress management, exercise and physical activity, and nutrition showed significant statistical differences before and after educational intervention. These observations suggest that improving health-promoting lifestyle components is associated with self-efficacy. A study conducted by Mirghafourvand also showed that there was a positive and significant correlation between self-efficacy and health-promoting lifestyle and its subdomains [35]. The study done by Amiri et al. showed a significant relationship between health and well-being and self-efficacy and health-enhancing lifestyle [36]; it is consistent with the present study. The results of these studies showed that lifestyle and its subdomains are closely associated with the self-efficacy of individuals. Also, after educational intervention, recognizing preventive behaviors, promoting health, and realizing that the ways to prevent the disease are cheap and simple, students found themselves able to perform preventive behaviors, and this increased their self-efficacy [37].

According to the results, the score of responsibility and self-fulfillment of individuals in the intervention group has increased significantly, while in the control group, there were no significant changes. In this regard, the study of Chenary et al. showed that the components of responsibility and self-actualization are associated with self-efficacy and change in one direction [38]. Ordun and Akün also showed that the flourishing of abilities and their application are directly associated with self-efficacy beliefs [39]. Another study done by Shcherbakova found that self-actualization was under the influence of a number of factors, one of the most important having positive beliefs about people's ability expressed in terms of self-efficacy [40]. The study of Charandabi et al. states that responsibility is associated with self-efficacy and emphasizes that responsibility is one of the characteristics of self-efficacious individuals [41]. A grounded theory study on female cancer survivors' experience of a psychological intervention for quality of life conducted by Durosini et al. showed that not only physical health but also self-actualization is one of the most important motivational reasons for healthy behaviors such as exercise [42].

The results of the present study also show a direct relationship between the component of interpersonal relationships and self-efficacy, as well as the effect of self-efficacy training on the component of interpersonal relationships. In their study, Saadat et al. stated that self-efficacy was associated with interpersonal relationships [43]. In a study, Siyez and Savi also pointed out the relationship between self-efficacy on the one hand and intimacy and empathy on the other hand, which emphasizes the existence of interpersonal relationships [44]. However, these studies have not stated that self-efficacy takes precedence over interpersonal relationships, or vice versa, because they are cross-sectional.

The present study shows that increasing self-efficacy improves stress management and researchers' intervention was able to improve teachers' stress control. Our findings support Bandura's (1988) suggestion that self-efficacy has important implications for people's health and well-being [45]. Although previous research has shown that self-efficacy may affect how employees respond to stressful situations [46–48], our study shows that self-efficacy structures can influence how teachers respond to real-world issues and promote stress management. However, the study done by Jurado et al. reported different results and showed that people with higher self-efficacy were under more stress. Researchers have interpreted that people with high self-efficacy are more responsible and therefore more stressed [49]. The results of the present study can be interpreted in such a way that people with high self-efficacy are better able to overcome problems and thus better control their stress [22]. The results of the study by Alipour et al., which supports the results of the present study, indicate that in order to achieve better results and positive effects on stress, anxiety, and depression in pregnant women, self-efficacy techniques can be used in the implementation of the training program [50].

Table 4 also shows that the changes observed in the intervention group in the component of physical activity and self-efficacy are in one direction. A study conducted by Andenæs et al. shows that physical activity and self-efficacy are associated. According to the results of these studies, we can say that physical activity can have a positive effect on a person's self-efficacy due to increasing the level of health [51]. We can also say that self-efficacy creates an important motivating force for people to perform regular physical activity. A study of Ostovarfar et al. found that self-efficacy is effective in altering behaviors associated with physical activity. People who have strong self-efficacy beliefs are more capable of controlling physical and situational problems [52]. The results of studies show a two-way correlation between self-efficacy and physical activity. Therefore, studies show that the advantage of physical activity is not limited to its physical effects, but these activities affect all aspects of health [52, 53].

Nutrition and self-efficacy were also directly associated with one another in this study. The study of Hall et al. is consistent with the present study and states that healthy nutrition leads to an improvement in a person's lifestyle and has a positive effect on a person's self-efficacy [54]. Schultz DP and Schultz SE state that proper nutrition is in the realm of health-related behaviors that are governed by self-efficacy beliefs [55]. Sharma et al. have conducted a study on nutritional self-efficacy aimed at assessing people's self-efficacy in consuming healthy foods. This study reported a close relationship between self-efficacy and nutrition [56]. Of course, given that teachers are among the most educated groups in society, they are aware of the importance of exercise and healthy nutrition, and as a result, the training sessions showed better results.

In general, we can say that increasing self-efficacy among teachers also promotes their lifestyle. Because there is a two-way relationship between the variables in this study, we can conclude that improving the health-promoting lifestyle components in teachers has improved their self-efficacy beliefs and behaviors.

Finally, we suggest that, due to the sensitive role of teachers as an effective human force in the development and evolution of society and their students' role modeling, the authorities should formulate policies, regulate educational interventions, and design strategies for promoting self-efficacy beliefs and promoting a healthy lifestyle for all teachers. We suggest that other methods and theories of behavior change be used in future studies to promote a healthy lifestyle. In this study, we have done educational intervention during two months; our suggestion is that future studies be conducted to investigate the continuity of behavior with longer follow-ups over several periods. This study also did not mention the effect of training in each session. This study was semiexperimental with limited sample size, so it is recommended that clinical trial studies be designed with a larger number of samples. Accordingly, we recommend that these cases be covered in future studies.

## Figures and Tables

**Table 1:** Summary of training sessions based on self-efficacy theory to improve health-promoting lifestyles.

First session	Health responsibility	Lectures to create an inner interest in increasing self-efficacy in a health-promoting lifestyle and that all people are responsible for their own health, along with providing educational pamphlets.
Second session	Stress management, self-actualization	“Reminding of direct experiences (skill experience)” as the main factor of self-efficacy and training stress management and self-fulfillment strategies.
Third session	Nutrition and physical activity	Using the “succession experiences” factor to help participants recall successful experiences. We asked successful people in the class (teachers) who have had successful experiences in promoting health behaviors (such as having a successful diet or exercising regularly) to share their experiences with others.
Fourth session	Stress management and self-actualization	Verbal and nonverbal persuasion through the practice of previous sessions to control stress and self-actualization strategies. Encouraging participants to build interpersonal relationships in the same session and giving positive feedback to the participants' discussions with each other.
Fifth session	Interpersonal relationships, health responsibility	Using interactive speech to create a positive mood by encouraging friendly relationships in participants to implement successfully a health-promoting lifestyle plan. We asked them to adjust their decisions to smaller, more accessible steps and hold themselves accountable for their decisions. We encouraged them with the successful completion of part of the program.
Sixth session	Physical activity, nutrition, stress management	We allowed individuals to express their thoughts and feelings when engaging in physical activity, following a healthy diet, and performing stress management practices, thus providing them with feedback on their condition.

**Table 2:** Demographic characteristics of the teachers under study.

Variable	Group	Domain	Mean	Standard deviation	^∗^ *P* value
Age	Experiment	23-48	33.40	5.68	0.61
	Control	23-49	32.83	6.46	

Number of deliveries	Experiment	0-4	1.31	1.22	0.88
	Control	0-4	1.28	1.18	

Work experience	Experiment	1-19	8.71	4.86	0.77
	Control	1-20	8.98	5.11	

Performing physical activities	Experiment	0-7	2.21	2.49	0.09
	Control	0-7	1.53	1.98	

^∗^Independent *t*-test.

**Table 3:** Mean and standard deviations of health-promoting lifestyle structures and self-efficacy in teachers under study.

Variable	Group	Before intervention (mean ± SD)	After intervention (mean ± SD)	*P* value
Spiritual growth and self-actualization	Intervention	25.90 ± 2.64	30.30 ± 5.97	*P* < 0.001
	Control	25.10 ± 2.53	25.38 ± 2.54	*P* = 0.63
	*P* value	*P* = 0.89	*P* = 0.001	

Health responsibility	Intervention	36.86 ± 7.12	41.38 ± 6.91	*P* < 0.001
	Control	36.03 ± 4.53	36.43 ± 5.94	*P* = 0.56
	*P* value	*P* = 0.44	*P* = 0.001	

Interpersonal relationships	Intervention	21.05 ± 2.16	24.11 ± 3.83	*P* < 0.001
	Control	20.96 ± 2.23	20.78 ± 2.64	*P* = 0.35
	*P* value	*P* = 0.83	*P* = 0.001	

Stress management	Intervention	15.71 ± 2.10	17.95 ± 2.71	*P* < 0.001
	Control	15.76 ± 2.13	15.76 ± 2.33	*P* = 0.74
	*P* value	*P* = 0.92	*P* = 0.001	

Exercise and physical activity	Intervention	18.93 ± 2.86	21.11 ± 3.90	*P* < 0.001
	Control	18.60 ± 2.89	18.53 ± 2.46	*P* = 0.32
	*P* value	*P* = 0.95	*P* = 0.001	

Nutrition	Intervention	19.06 ± 2.49	20.81 ± 3.56	*P* = 0.004
	Control	19.10 ± 2.54	19.06 ± 2.49	*P* = 0.44
	*P* value	*P* = 0.91	*P* = 0.002	

Overall lifestyle score	Intervention	137.53 ± 9.41	155.68 ± 24.58	*P* < 0.001
	Control	136.53 ± 8.15	136.96 ± 8.79	*P* = 0.51
	*P* value	*P* = 0.53	*P* = 0.001	

Self-efficacy	Intervention	53.86 ± 6.51	62.53 ± 8.67	*P* < 0.001
	Control	53.75 ± 6.63	53.86 ± 6.51	*P* = 0.12
	*P* value	*P* = 0.92	*P* = 0.001	

**Table 4:** Pearson correlation coefficient between subscales of lifestyle and self-efficacy in teachers.

Variable	Spiritual growth and self-actualization	Health responsibility	Interpersonal relationships	Stress management	Physical activity	Nutrition	Overall lifestyle	Self-efficacy
Spiritual growth and self-actualization	1	0.870	0.913	0.797	0.591	0.798	0.927	0.567
Health responsibility	0.870	1	0.919	0.856	0.534	0.809	0.933	0.941
Interpersonal relationships	0.913	0/919	1	0.881	0.677	0.780	0.954	0.918
Stress management	0.797	0.856	0.881	1	0.854	0.956	0.957	0.686
Physical activity	0.591	0.534	0.677	0.854	1	0.848	0.775	0.647
Nutrition	0.798	0.809	0.780	0.956	0.848	1	0.928	0.763
Overall lifestyle	0.927	0.933	0.954	0.957	0.775	0.928	1	0.853
Self-efficacy	0.567	0.941	0.918	0.686	0.647	0.763	0.853	1

## Data Availability

The statistical data used to support the findings of this study are included within the article.
